# The Preoperative Planning, Design, and Execution of the Freestyle Propeller Flap: A Detailed Description and the Case Series

**DOI:** 10.1055/a-2620-3297

**Published:** 2025-07-23

**Authors:** Hyung Bae Kim, Seong John Han, Joon Pio Hong, Hyunsuk Peter Suh

**Affiliations:** 1 Department of Plastic and Reconstructive Surgery, Asan Medical Center, University of Ulsan College of Medicine, Seoul, Republic of Korea

**Keywords:** propeller flap, soft tissue reconstruction, trunk, lower extremities

## Abstract

**Background:**

Propeller flap reconstruction has been widely utilized in soft tissue reconstruction due to its versatility and aesthetic outcomes. However, technical challenges and the risk of complications persist. This study aims to provide detailed guidelines on the preoperative planning, intraoperative considerations, and execution of propeller flap surgery to reduce complications.

**Methods:**

A retrospective review was conducted on 20 consecutive patients undergoing propeller flap reconstruction between January 2018 and December 2020. Preoperative planning involved computed tomography (CT) angiography and color Doppler ultrasound. Flap designs prioritized perforator proximity (<3 cm from the defect), vessel axiality, and tissue laxity assessed by skin pinch tests. Surgical techniques including pedicle skeletonization, flap elevation, rotation, and inset were meticulously followed.

**Results:**

No total flap loss occurred. Partial flap loss was observed in one case (5%). Two flaps (10%) exhibited venous congestion, which resolved following leech therapy without necrosis. Defects were predominantly located on the trunk (80%), with malignancy as the primary cause (55%). Mean follow-up duration was 432 days.

**Conclusions:**

Careful preoperative planning and adherence to meticulous surgical techniques can significantly reduce complications in propeller flap reconstruction. This structured approach offers a reliable framework, particularly beneficial for surgeons less familiar with propeller flap techniques.

## Introduction

Propeller flap reconstructions have been performed in various body areas with adequate perforators for soft tissue reconstruction. 1 2 3 4 The major advantages of the propeller flap include less pedicle dissection during flap elevation, faster operation times, no need for microsurgery, and superior aesthetic results. 5 6 7 8 9 10 As long as the surrounding tissue is preserved, the propeller flaps can be considered for covering the soft tissue defect.

Despite their increasing versatility and reliability, performing propeller flap reconstruction without complications is challenging due to various obstacles. 11 12 13 14 One of the most common reasons for failure in a propeller flap is inappropriate indication. Even though the concepts and fundamental principles have been reported, propeller flaps are often attempted in cases that are not ideal. 10 11 In addition to inappropriate indications, errors and mistakes in the surgical procedures at each step can result in complications such as arterial and venous insufficiency and flap failure.

This article describes the technical details of designing and performing propeller flap reconstructions, including preoperative, intraoperative, and surgical considerations. A retrospective review of propeller flaps has been conducted, and cases of propeller flaps are presented. This article will be beneficial for young surgeons who are not familiar with propeller flaps.

## Methods

This retrospective study, approved by the Institutional Review Board (No. 2025-0099), involved 20 consecutive patients who underwent propeller flap reconstruction between January 2018 and December 2020 at a single center. Demographic data, flap information, and reconstructive outcome data were collected. Written informed consent was obtained from all patients for the publication of their clinical photographs.

### Step-by-Step Approach of the Propeller Flap

**Video 1** A step-by-step approach to propeller flap reconstruction.


The video provides a step-by-step summary of the propeller flap procedure ( Video 1 ).

### Preoperative Planning Using Computed Tomography Angiography and Color Doppler Ultrasound

The authors used computed tomography (CT) angiography and color Doppler ultrasound for preoperative evaluation. CT angiography can identify a suitable perforator around the defect. Color Doppler ultrasound, with its real-time imaging, can directly mark the vessels and provide hemodynamic data, including flow velocity, vessel diameter, and the axis of the perforator course. 15 16 17 18 19 For the propeller flap, the intraflap perforator course (axis) is crucial for designing the flap to maximize circulation. High-frequency color Doppler ultrasound can easily identify and preoperatively mark the perforator axis. 18

### Perforator Identification and Considerations in Flap Design

**Fig. 1:**
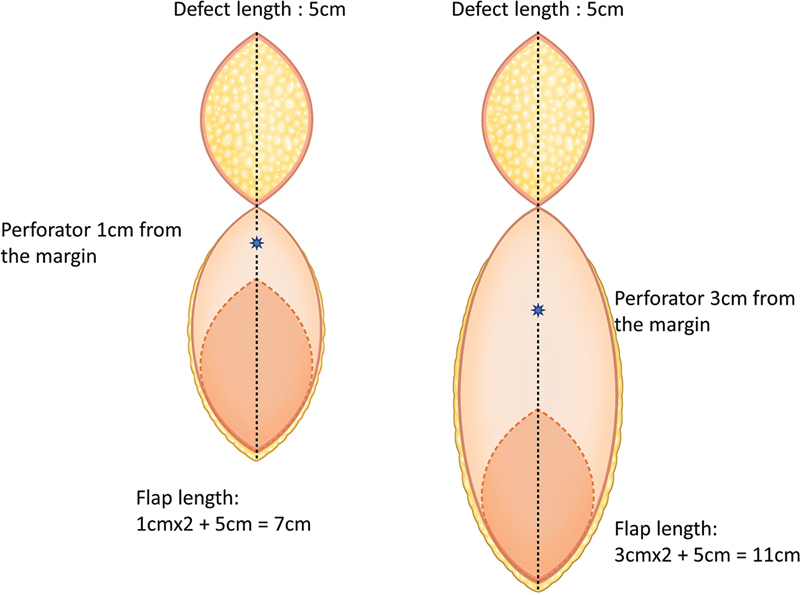
Distance of the perforator from the defect and flap design **.**

**Fig. 2:**
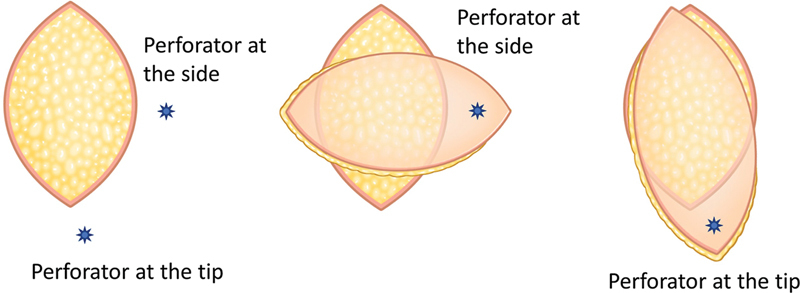
Location of the perforator and flap design (tip and side).

**Fig. 3:**
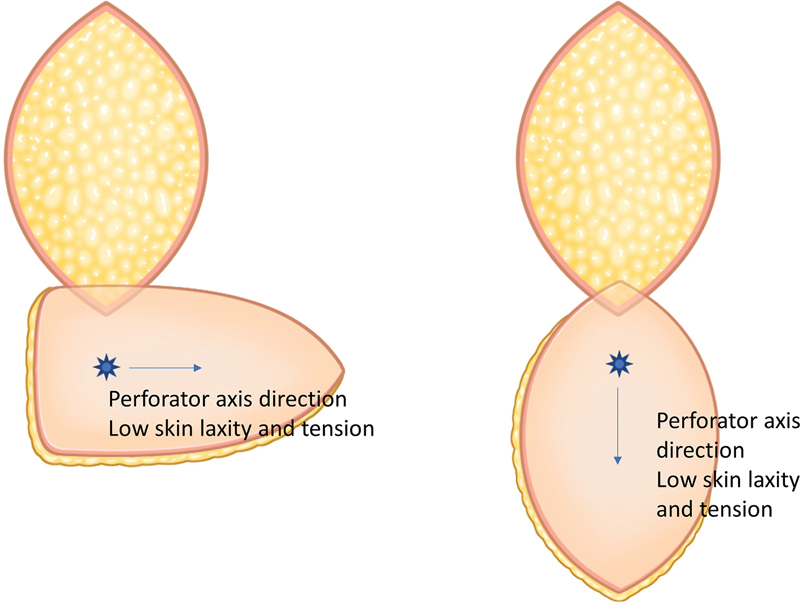
Skin laxity and the axis of the perforator and flap design.

Using proper retractors, perforators were dissected above the deep fascia and identified directly from the margin of the defect. An additional incision is not usually needed if a handheld Doppler is used to identify the perforators. The authors then chose the perforator with good blood flow, one <3 cm from the defect, and one near the tip of the defect. If the perforator is 1 cm away from the margin, the flap length is increased to twice as long as 2 cm ( Fig. 1 ). A perforator closer to the tip than the side is advantageous for effectively covering the defect ( Fig. 2 ). For the flap size, a flap margin over 10 cm from the perforator may result in partial necrosis. 20 When determining the axis of the flap, the axis of the perforator course and tissue laxity should be considered. Good axiality of the perforator can provide more blood supply to the area most distant from the perforator, minimizing partial necrosis of the flap. Skin laxity is considered when determining not only the flap direction but also the flap width for primary donor site closure. Skin laxity is evaluated by a skin pinch test ( Fig. 3 ). Flap design should comprehensively consider the perforator position, its relation with the tip position, perforator to margin distance, and the axis of the flap.

### Considerations in Locating the Perforator in the Propeller Flap

**Fig. 4:**
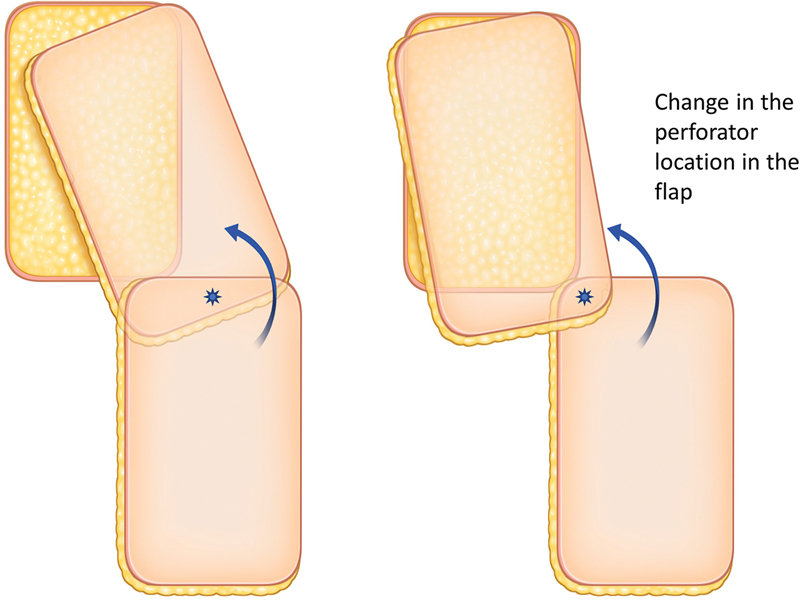
Location of the perforator in the propeller flap.

When designing propeller flaps, the question arises of where to position the perforators. Typically, the perforator is placed in the middle and distal part of the flap. However, it is not always necessary to place it in the middle. More importantly, the flap should be designed to fill all defects when rotated. Sometimes, it may be more advantageous to place the perforator on one side to cover the defect, as shown in Fig. 4 .

### Flap Elevation and Pedicle Skeletonization

**Fig. 5:**
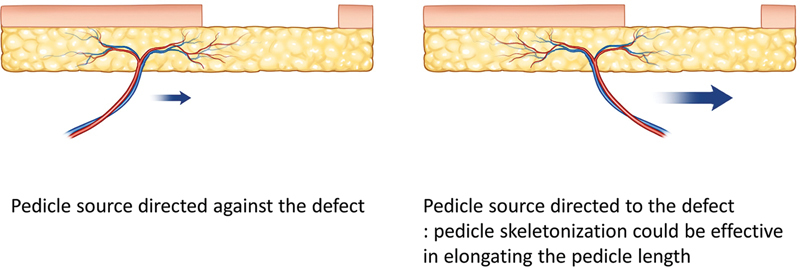
Skeletonization of the perforator and origin of the perforator.

After designing the flap, flap elevation is performed from the flap's margin. About 2 cm of pedicle skeletonization is generally sufficient for rotation. If the pedicle source is directed toward the defect, pedicle skeletonization can effectively elongate the pedicle length. If the origin of the pedicle source is directed away from the defect, pedicle skeletonization has minimal effect on elongating the pedicle length ( Fig. 5 ).

### Flap Inset and Rotation

After flap elevation, rotation and flap inset are performed. Rotation is done in a direction less than 180 degrees, and the state of the pedicle is evaluated. Flap perfusion should be assessed after rotation, and two things must be checked to determine if perfusion is inadequate. First, evaluate whether excessive tension is on the flap and perform the inset again. Second, after rotating in the opposite direction, reassess perfusion. A hemodynamic study using color duplex ultrasound can help determine the direction of rotation (clockwise or counterclockwise). 21

### Considerations in Arterial and Venous Insufficiency after Flap Inset

After inset, immediate arterial or venous insufficiency can occur. Careful checking of the pedicle vessels is necessary. If pedicle compromise includes kinking, compression, or stretching, the surgeon should identify the reasons for the compromise and reduce the mechanical burdens on the pedicle vein. If venous congestion is limited to the flap margin, leeches can be applied, and partial congestion may resolve after a few days. If venous congestion persists despite efforts to reduce pedicle compromise, delayed flap inset should be considered. Delayed flap inset involves returning the propeller flap to the original donor site and reinserting it after 1 to 2 weeks. This procedure is based on the “delay phenomenon” of the flap. 22

## Results

The mean patient age was 52.8 years, with a mean follow-up period of 432 days. The defect was primarily located on the trunk (80%) and lower extremities (20%). The cause of the defect was malignancy in 55% of patients and pressure sores in 45% ( Table 1 ). The mean pedicle distance from the margin was 2.4 cm (range, 1–4 cm). There was no total flap loss. The partial flap loss rate was 5% ( *n* = 1). Two flaps (10%) experienced venous congestion without partial necrosis after leech application ( Table 2 ).

**Table 1:** Demographic data

Characteristics		Value
Age (years), mean (range)		52.8 (18–86)
Sex, *n*	Male	11
	Female	9
Comorbidity, *n* (%)	HTN	7 (35%)
	DM	4 (20%)
	Other	1 (5%)
Smoker, *n* (%)	Current smoker	6 (30%)
Etiology of the defect, *n* (%)	Malignancy	11 (55%)
	Pressure sore	9 (45%)
Location of the defect, *n* (%)	Trunk	16 (80%)
	Lower extremity	4 (20%)
Follow-up (days), mean (range)		432 (62–1,260)

Abbreviations: DM, diabetes mellitus; HTN, hypertension.

**Table 2:** Flap information and reconstructive outcomes

Characteristics		Value
Defect size (cm ^2^ ), mean (range)		82.4 (25–180)
Flap size (cm ^2^ ), mean (range)		130.4 (72–276)
Pedicle distance from the margin (cm), mean (range)		2.4 (1–4)
Supercharged, *n* (%)		2 (10%)
Revision, *n* (%)		1 (5%)
Complication, *n* (%)	Total loss	0 (0%)
	Partial loss	1 (5%)
	Venous congestion (leech used)	2 (10%)
	Donor site complication	0 (0%)

**Fig. 6:**
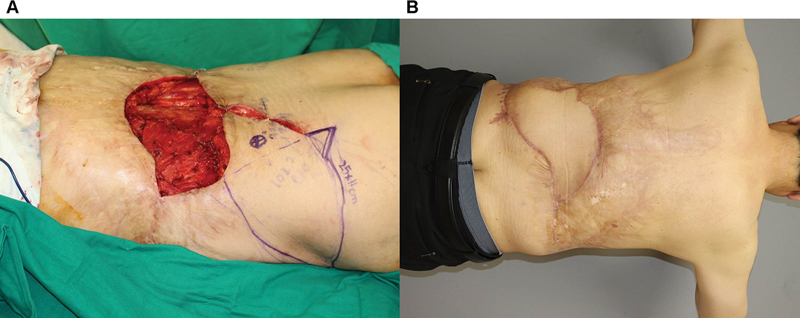
Case of a patient with Marjolin's ulceration on his back. ( **A** ) Preoperative design after wide excision (clockwise 90° rotation). ( **B** ) Postoperative clinical photography in outpatient clinic (counterclockwise 90° rotation).

**Fig. 7:**
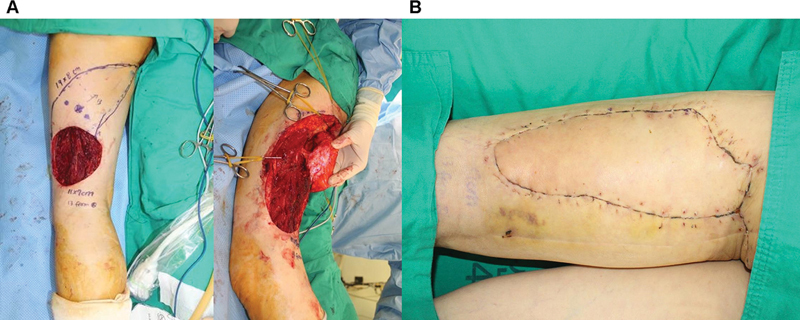
Case of a patient with myxofibrosarcoma on his left thigh. ( **A** ) Propeller flap design after excision of myxofibrosarcoma. ( **B** ) Postoperative clinical photography in outpatient clinic.

**Fig. 8:**
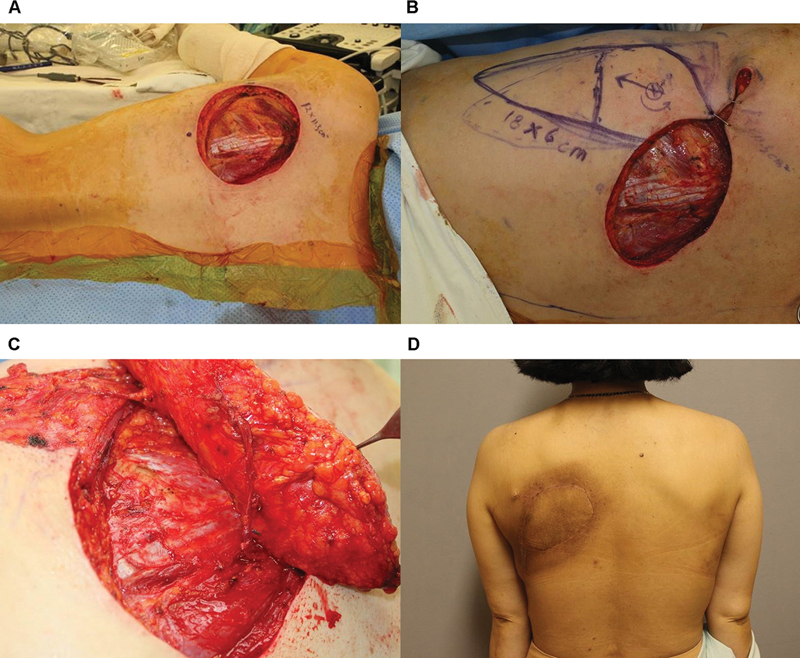
Case of a patient with dermatofibrosarcoma protuberance on her left upper back. ( **A** ) Defect after wide excision. ( **B** ) Propeller flap design after based on perforator near defect. ( **C** ) Pedicle noted after flap elevation. ( **D** ) Postoperative clinical photography in outpatient clinic.

**Fig. 9:**
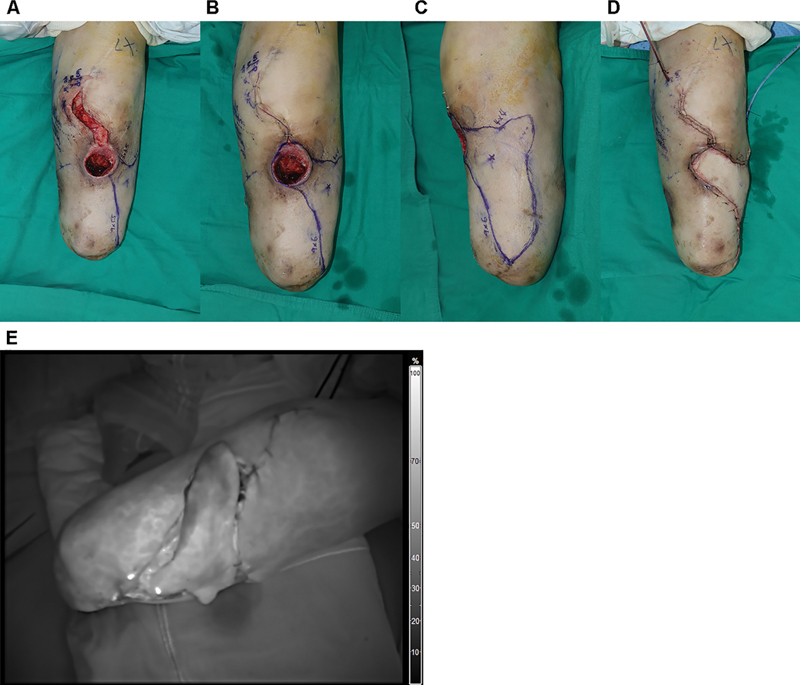
Case of a patient with chronic osteomyelitis on his left lower leg. ( **A-C** ) Propeller flap design near the defect on lower leg. ( **D** ) Immediate postoperative photography. ( **E** ) Post-insetting ICG angiography showed good perfusion.

Representative case examples are shown. Figure 6 shows a 64-year-old male patient with Marjolin's ulceration on his back ( Fig. 6A ). A 25 × 10 cm propeller flap based on the axiality of the perforator and skin laxity was designed and elevated. At 12 months, the patient had a good contour of the flap without complications ( Fig. 6B ). Figure 7 shows a 44-year-old male patient with myxofibrosarcoma on his left thigh ( Fig. 7A ). A 15 × 7 cm propeller flap based on the axiality of the perforator and skin laxity was designed and elevated. At 1 month, the patient had a good contour of the flap without complications ( Fig. 7B ). Figure 8 shows a 43-year-old female patient with dermatofibrosarcoma protuberance in her left upper back. A 12 × 11.5 cm defect was created after a wide excision ( Fig. 8A ). An 18 × 6 cm propeller flap based on the axiality of the perforator was designed ( Fig. 8B ). An arterial supercharge was performed to increase blood flow to the distal area ( Fig. 8C ). At 1 year, the patient had a good contour of the flap without complications, even after radiation therapy ( Fig. 8D ). Figure 9 presents a 56-year-old male patient with chronic osteomyelitis on his left lower leg after below knee amputation ( Fig. 9A ). A 9 × 6 cm propeller flap was designed to cover a 5 × 6 cm defect on the anterior leg ( Fig. 9B– 9D ). Adequate perfusion of the flap after elevation was confirmed with ICG angiography ( Fig. 9E ).

## Discussion

By understanding the anatomical knowledge of the perforasome, propeller flap surgery can theoretically be used in various parts of the body in different ways. 23 24 25 To achieve successful reconstruction, surgeons must understand the surgical details to focus on during planning, designing, and postoperative management. However, few articles describe the surgical details of performing the propeller flap.

This article emphasizes the surgical details of performing the propeller flap. Designing and planning the flap before surgery is crucial because the propeller flap features a perforator that is positioned laterally and rotated. If surgeons do not pay attention to each step, complications such as partial necrosis, venous congestion, and incomplete coverage of the defect are likely.

The propeller flap does not have a specific indication but is an excellent reconstructive option when certain conditions outlined in the text are met. First, an appropriate perforator within 3 cm of the defect must be evaluated and confirmed through imaging modalities such as CT angiography or color Doppler ultrasound. Additionally, the donor site must be assessed using a pinching test to ensure sufficient surrounding tissue is available for transfer. A comprehensive evaluation is essential to determine whether the propeller flap is optimal. If deemed unsuitable, alternative reconstructive options should be considered before proceeding with surgery.

The major complications associated with the propeller flap are arterial insufficiency, venous congestion, and partial or total necrosis of the flap. Among these, venous congestion is generally believed to be the most common complication in propeller flap surgery, occurring in 5 to 11% of cases. 26 Given that veins have thinner and more pliable walls than arteries, venous congestion is more easily related to pedicle compromise, including compression, kinking, and stretching. Care should be taken during pedicle dissection to avoid damaging the veins and to check the pedicle vein for any compression, kinking, or stretching.

Venous congestion management varies depending on its timing. If congestion arises immediately after flap elevation, supercharging should be attempted to enhance venous outflow. If unsuccessful, leech therapy and systemic heparinization may be employed. In cases where congestion occurs after flap rotation, additional pedicle dissection should be performed to identify and correct kinking or excessive stretching. If these measures fail, the flap can be derotated and temporarily returned to the donor site, with re-insetting attempted approximately 5 days later. This approach leverages the delayed phenomenon, which improves flap perfusion through angiogenesis and vascular adaptation.

Partial flap necrosis is another frequent complication in propeller flaps. 11 13 27 To reduce partial flap necrosis, one of the most challenging considerations in designing the propeller flap is determining the location of the perforator from the flap margin. The authors described a perforator less than 3 cm from the defect and a perforator near the tip of the defect. However, the possibility of partial necrosis increases if the perforator is too close to the flap margin and far from the distal area. According to a large-volume report on partial necrosis of the perforator flap, a distance of 10.25 cm from the perforator significantly increases the possibility of partial necrosis. 20 Partial necrosis of the propeller flap usually occurs in the most distal area, which is crucial for covering the defect. Surgeons should consider this point when designing large propeller flaps. ICG angiography after harvesting the flap can help predict the occurrence of partial necrosis. 28

Another consideration in the propeller flap is radiation after cancer resection. The biggest difference between the free flap and the propeller flap in the reconstruction of sarcoma resection is that the resection margin may move away from its original position during flap rotation by as much as 7 to 8 cm from the original tumor margin. 29 Although there is a report that there were no significant differences in disease-free survival between patients who underwent propeller flap reconstruction and those who underwent free flap reconstruction, a multidisciplinary approach and discussion are needed to determine the accurate planning target volume of adjuvant radiotherapy.

There are several limitations in this study. First, it is designed as a retrospective study, not a prospective one. Second, the sample size is relatively small. However, this article aims to describe the surgical details and share cases of the propeller flap.

### Conclusion

In this article, we described the proper planning and technical details. Preoperative planning and proper design are crucial in reducing complications and ensuring successful surgery. This article will be beneficial for surgeons who are not familiar with propeller flaps.
